# Supplementary material for: Chiral supramolecular assembly to enhance the magneto-optical rotation of organic materials
Source: Nat Commun. 2025 Nov 3;16:9684. doi: 10.1038/s41467-025-64663-w (PMC12583486; doi:10.1038/s41467-025-64663-w)
Supplement: Supplementary file 1 — Supplementary material [file 41467_2025_64663_MOESM1_ESM.pdf]

Contents

Suppl. Note 1. Faraday rotation instrument and short discussion of artifacts......... 1

Suppl. Note 2. Detailed theory of detection.. 4

Suppl. Note 3. Film thickness measurements 6

Suppl. Note 4. Verdet spectra acquired at the Diamond Light Source 8

Suppl. Note 5. Magnetic field dependence.. 9

Suppl. Note 6. Thickness dependency of the Verdet constant and further discussion of the supramolecular structure.. 10

Suppl. Note 7. Fits to a simple model of excitonically coupled dimers................. 12

Suppl. Note 8. Figure of Merit (FoM) for Faraday rotation 12

Suppl. Note 9. Polarization of light after passing through the film 12

Supplementary references.. 14

Suppl. Note 1. Faraday rotation instrument and short discussion of artifacts

Initial measurements of enantiopure TA films revealed unexpected absorption line shapes and an apparent chirality-dependent sign change of θ_F. This is not in accordance with the established model for MOA. As a result, we carefully examined the instrument setup and underlying theory.

The magnetic circular birefringence (MCB) spectra were recorded on a home-built magneto-optical spectrometer, which has been described previously (Supplementary Figure *1*).[1] Balanced photodetectors receive orthogonal light beams (I_A and I_B) after linearly polarized light has passed through a sample and subsequently been split by a Wollaston prism (Supplementary Figure *1*). When no magnetic field is applied or the MCB is negligible, the light intensity of both beams is equivalent ($I_A = I_B$), and the MCB is either 0 or below the detection limit:

$$I_A - I_B \approx \mathrm{CB}\, I \qquad (Supp.\,eq.\,1)$$

We note that this expression includes the total intensity of light (I) incident upon the Wollaston prism, which should equal the summation of I_A and I_B. To measure the CB independent of the intensity, we consider the dimensionless ratio:

$$\frac{I_A - I_B}{I_A + I_B} \approx \mathrm{CB} \qquad (\mathrm{Supp.\,eq.\,2})$$

If the CB is sufficiently small, we can approximate $I_A \approx I_B$, i.e. the denominator above satisfies $I = I_A + I_B \approx 2I_A \approx 2I_B$.

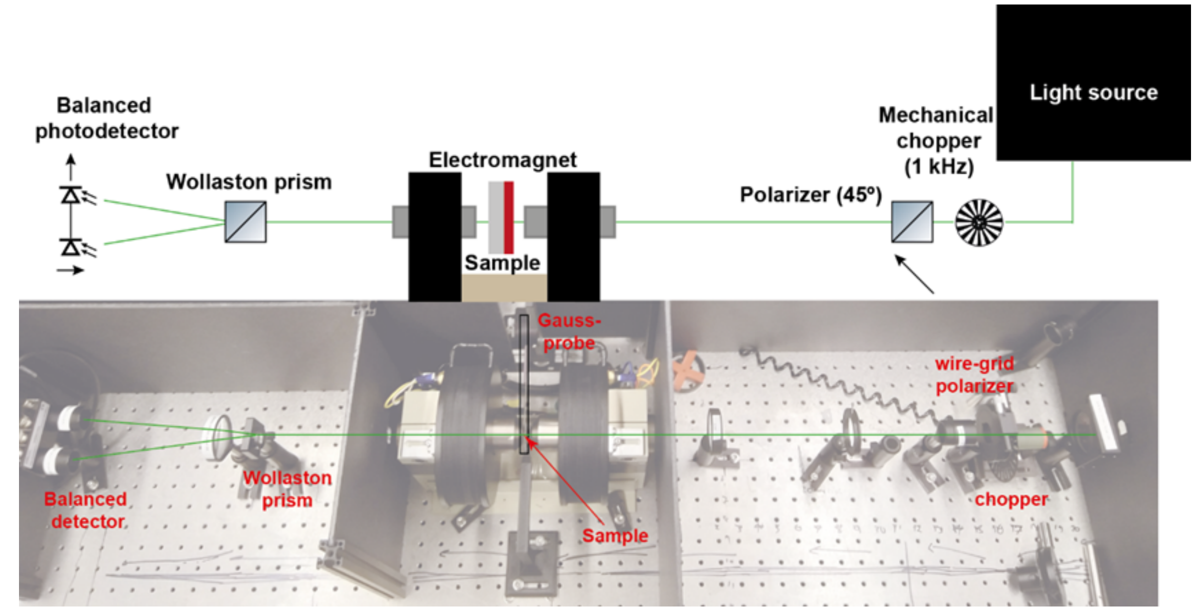

Supplementary Figure 1: The home-built Faraday rotation spectrometer.

This approximation holds when molecular systems studied have very small CB (e.g. they are achiral or have very weak chiroptical activity), which includes almost all molecular systems previously characterised. For molecular systems with very large circular dichroism (CD) and very large CB (e.g. the TA chiral polymer films considered here) this approximation fails. Depending on the handedness of the supramolecular assembly, I_A or I_B will be significantly larger while the intensity of the other orthogonal beam will be significantly lower. Since I_A (or I_B) is used for both the calculations of total light intensity (I) and the MCB ($I_A – I_B$), its erroneous reading led to inaccurate UV-Vis and MCB spectra. Depending on which photodetector channel is used for the light intensity approximation (Monitor+ or Monitor-), several changes to the spectra are observed (Supplementary Figure 2), including fluctuations to the UV-Vis absorption spectra and artifactually high Verdet constant values (*V*). The artifacts also resulted in a shift in the wavelength and sign of peak *V* depending on the handedness of the supramolecular assembly (Supplementary Figure 2). To validate this hypothesis, a reported achiral magneto-optical material, decamethylferrocene radical cation,[2] was measured while the detection pathways (Monitor+ and Monitor-) were switched. Identical UV-Vis and MCB spectra were measured (Supplementary Figure 3), demonstrating that the large CB of the studied molecular systems caused the artifacts.

We modified the instrument to include a summing amplifier (SIM 980) to account for the signals from both photodetectors (Supplementary Figure 2). The instrument was calibrated using commercial inorganic Faraday rotators (BK7 and TGG, Thorlabs).

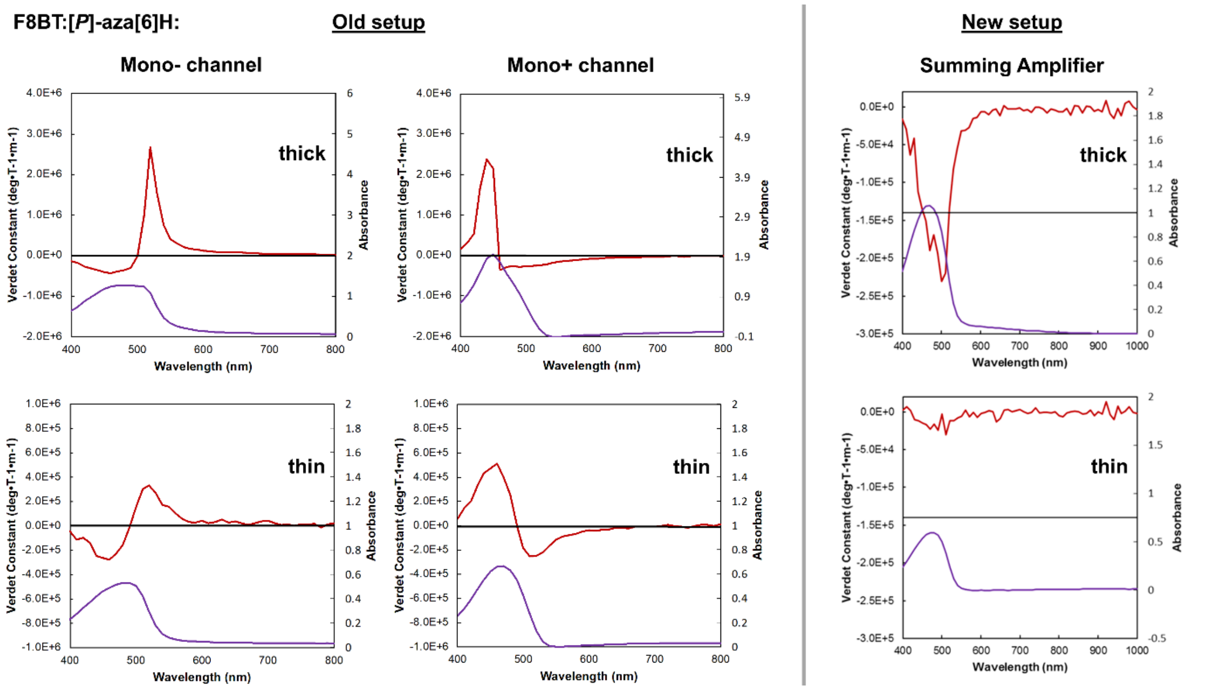

Supplementary Figure 2: MCB and UV−vis spectra of **F8BT:[*P*]-aza[6]H** measured on the "old" setup (left) compared with spectra on the new setup (right).

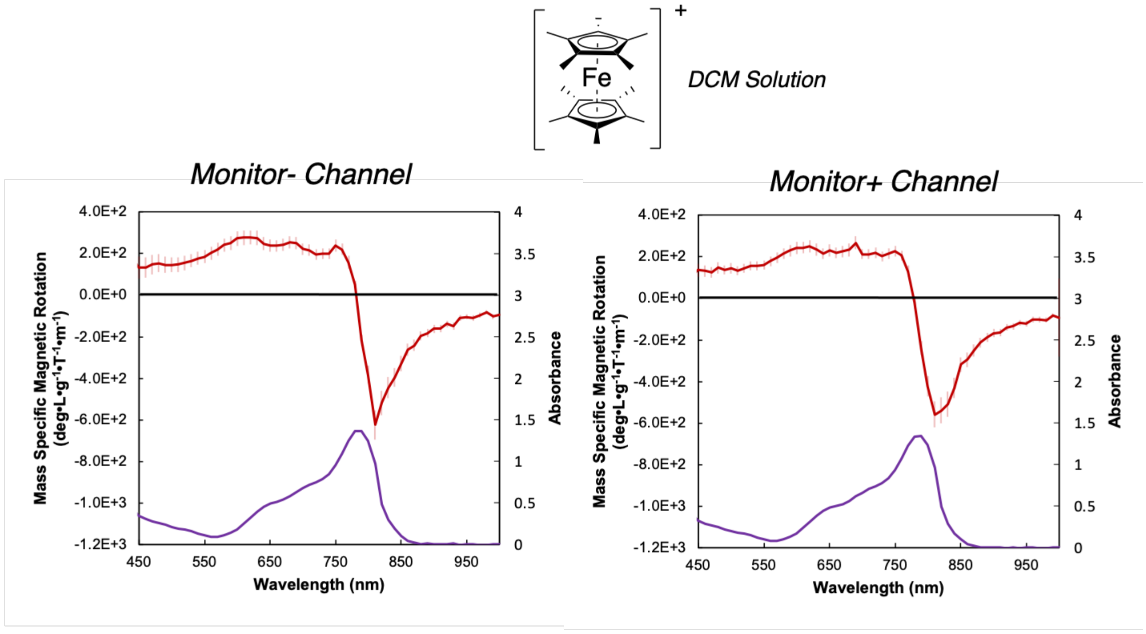

Supplementary Figure 3: MCB and UV−vis spectra of decamethylferrocene radical cation. The sample is a 3×10^{-3} M solution of a 1:1 mixture of decamethylferrocene and nitrosonium tetrafluoroborate in dichloromethane.

Suppl. Note 2. Detailed theory of detection

Here we present a detailed theory of the two detection approaches described above, namely detection with and detection without the summing amplifier. The theory emphasises how the latter approach can give artifacts for the MCB of chiral samples with large NOA. For the sake of brevity, we use Barron's formalism and notation.[3]

Suppose that light with initial intensity I_0, degree of polarisation P_0, ellipticity η_0 and azimuth θ_0 propagates through the sample to give $I = I_0 + \Delta I$, $P = P_0 + \Delta P$, $\eta = \eta_0 + \Delta\eta$ and $\theta = \theta_0 + \Delta\theta$ as the parameters of the light incident upon the Wollaston prism. Let detector A (e.g. Monitor+) measure the intensity I_A of vertically polarised light emerging from the Wollaston and detector B (e.g. Monitor–) measure the intensity I_B of horizontally polarised light:

$$I_A = \frac{I}{2}[1 + P\cos(2\eta)\cos(2\theta)] \qquad \text{(Supp. eq. 3)}$$

$$I_B = \frac{I}{2}[1 - P\cos(2\eta)\cos(2\theta)] \qquad \text{(Supp. eq. 4)}$$

For our dimensionless ratio, we consider one of the following:

$$\Delta = \frac{I_A - I_B}{I_A + I_B} = P\cos(2\eta)\cos(2\theta) \qquad (Supp. eq. 5)$$

$$\Delta' = \frac{I_A - I_B}{2I_{\frac{A}{B}}} = \frac{P\cos(2\eta)\cos(2\theta)}{1 \pm P\cos(2\eta)\cos(2\theta)} \qquad (Supp. eq. 6)$$

where Δ (unprimed) describes detection *with* the summing amplifier and Δ' (primed) describes detection *without*. The upper sign in Δ' corresponds to the case where the A detection channel is used for the denominator and the lower sign corresponds to the case where the B detector is used.

For the sake of concreteness let us suppose now that the light incident upon the sample has perfect linear polarisation with an azimuth of minus forty-five degrees
($P_0 = 1$, $\eta_0 = 0$ and $\theta_0 = -45°$), giving

$$\Delta \approx (1 + \Delta P)\cos(2\Delta\eta)\sin(2\Delta\theta) \qquad (Supp. eq. 7)$$

$$\Delta' \approx \frac{(1 + \Delta P)\cos(2\Delta\eta)\sin(2\Delta\theta)}{1 \pm (1 + \Delta P)\cos(2\Delta\eta)\sin(2\Delta\theta)} \qquad (Supp. eq. 8)$$

If we assume that the sample induces negligible changes in the degree of polarisation and only *small* changes in the ellipticity and/or azimuth ($\Delta P \approx 0$, $|\Delta\eta| \ll 1$ and $|\Delta\theta| \ll 1$), these equations reduce immediately to

$$\Delta \approx \Delta' \approx 2\Delta\theta \qquad (Supp. eq. 9)$$

Thus, the signal is proportional to the angle of optical rotation $\Delta\theta$ for either detection approach, as desired.

What happens when we have a sample with large NOA and the above assumptions fail? Let us focus on the case of a uniaxial sample that induces negligible changes in the degree of polarisation ($\Delta P \approx 0$) but potentially large changes in the ellipticity and azimuth of the form

$$\Delta\theta \approx \chi l + VlB_z \qquad (Supp. eq. 10)$$

$$\Delta\eta \approx \tan^{-1}[\tanh(\chi' l + V' lB_z)] \qquad (Supp. eq. 11)$$

where χl describes the NCB ("natural" circular, B = 0, birefringence) of the sample, VlB_z describes the MCB, $\chi' l$ describes the NCD and $V' lB_z$ describes the MCD, l = sample thickness, V = Verdet constant, V' being the absorptive counterpart of V and B_z being the static magnetic field component in the direction of propagation. Thus:

$$\Delta \approx \cos\{2\tan^{-1}[\tanh(\chi' l + V' lB_z)]\}\sin[2(\chi l + VlB_z)] \qquad (Supp. eq. 12)$$

$$\Delta' \approx \frac{\cos\{2\tan^{-1}[\tanh(\chi' l + V' lB_z)]\}\sin[2(\chi l + VlB_z)]}{1 \pm \cos\{2\tan^{-1}[\tanh(\chi' l + V' lB_z)]\}\sin[2(\chi l + VlB_z)]} \qquad (Supp. eq. 13)$$

giving

$$\left.\frac{\partial\Delta}{\partial B_z}\right|_{B_z=0} \approx 2Vl\cos(2\chi l)\cos\{2\tan^{-1}[\tanh(\chi' l)]\} - 2V' l\,\mathrm{sech}(2\chi' l)\sin(2\chi l)\sin\{2\tan^{-1}[\tanh(\chi' l)]\} \qquad (Supp. eq. 14)$$

$$\left.\frac{\partial\Delta'}{\partial B_z}\right|_{B_z=0} \approx \frac{2Vl\cos(2\chi l)\cos\{2\tan^{-1}[\tanh(\chi' l)]\}[1+\tanh^2(\chi' l)]}{(1 \pm \cos\{2\tan^{-1}[\tanh(\chi' l)]\}\sin(2\chi l))^2[1+\tanh^2(\chi' l)]} - \frac{2V' l\,\mathrm{sech}^2(\chi' l)\sin(2\chi l)\sin\{2\tan^{-1}[\tanh(\chi' l)]\}}{(1 \pm \cos\{2\tan^{-1}[\tanh(\chi' l)]\}\sin(2\chi l))^2[1+\tanh^2(\chi' l)]} \qquad (Supp. eq. 15)$$

The apparent *V* follows as

$$V_{\text{apparent}} = \frac{1}{2l}\frac{\partial\Delta}{\partial B_z}\bigg|_{B_z=0} \approx V \qquad (Supp.\,eq.\,16)$$

$$V'_{\text{apparent}} = \frac{1}{2l}\frac{\partial\Delta'}{\partial B_z}\bigg|_{B_z=0} \approx V(1 \mp 4\chi l) \qquad (Supp.\,eq.\,17)$$

#

to first order in l. Evidently, V_{apparent} gives a good approximation to chirality-independent V, as desired. Note also that V_{apparent} has no term linear in l, suggesting that the observed variation with film thickness is legitimate. In contrast V'_{apparent} suffers from an instrumental artefact that has opposite signs for Monitor+ and Monitor- detection channels (via $\mp$) or handedness of the supramolecular assembly (via χ).

Suppl. Note 3. Film thickness measurements

Film thickness was determined using cross-sectional SEM (JEOL 6010LA). Thin films were coated with Au-Pd (Denton Desk V Sputter Coater) prior to measurement.

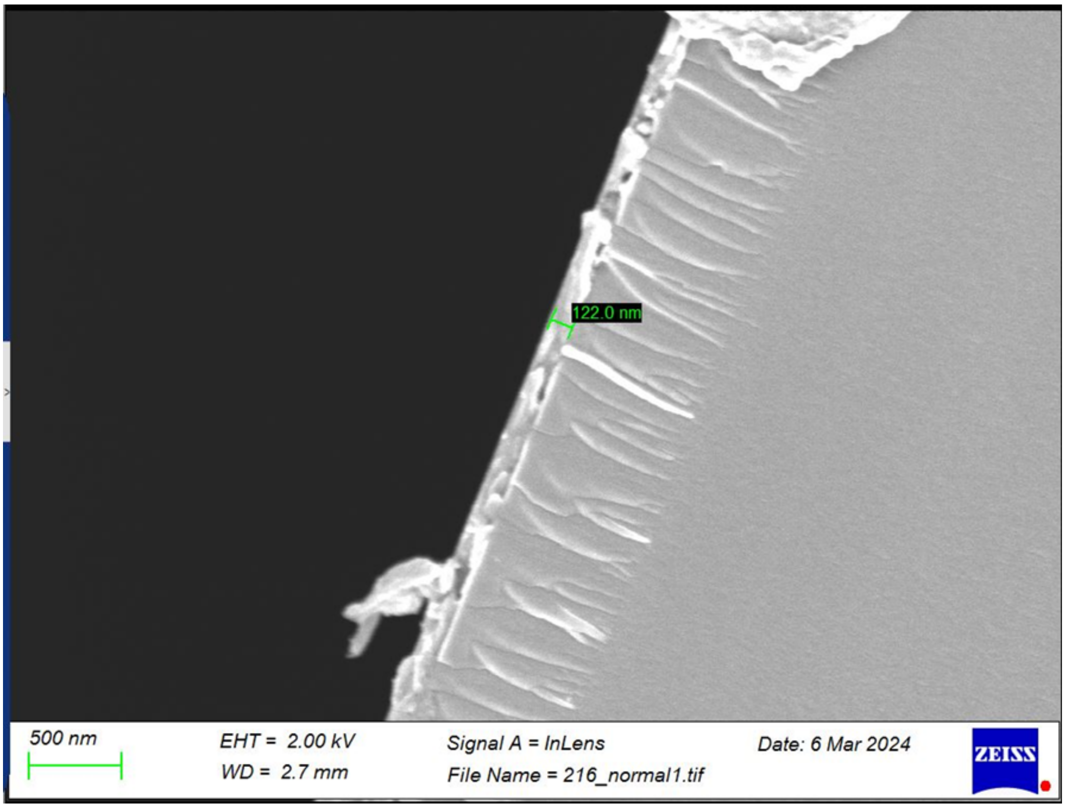

Supplementary Figure 4: Cross sectional SEM image of **F8BT: [*P*]-aza[6]H** (122 nm).

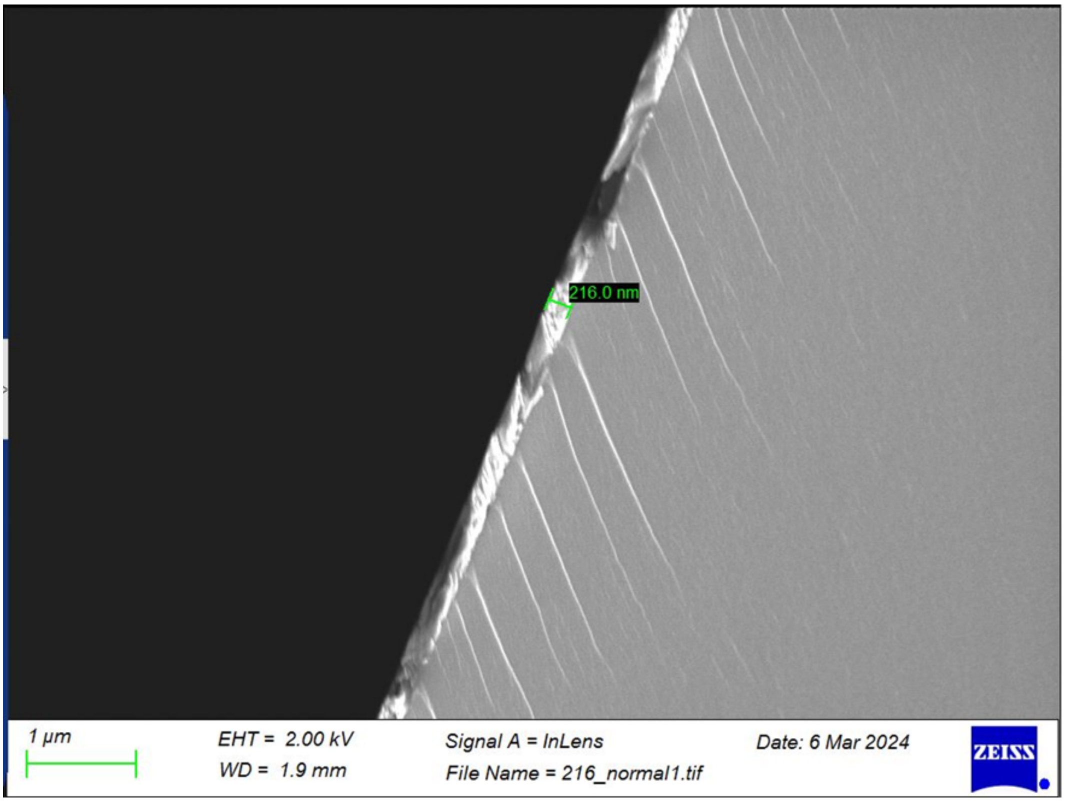

Supplementary Figure 5: Cross sectional SEM image of **F8BT: [*P*]-aza[6]H** (216 nm).

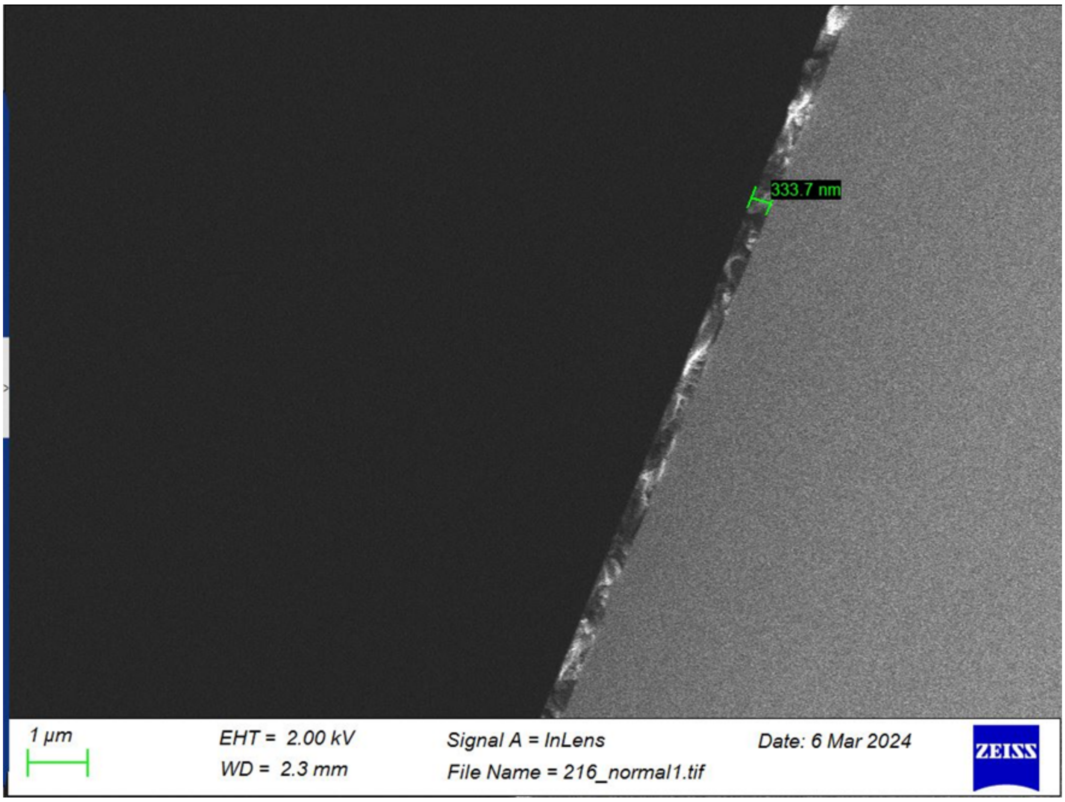

Supplementary Figure 6: Cross sectional SEM image of **F8BT: [*P*]-aza[6]H** (333 nm).

Suppl. Note 4. Verdet spectra acquired at the Diamond Light Source

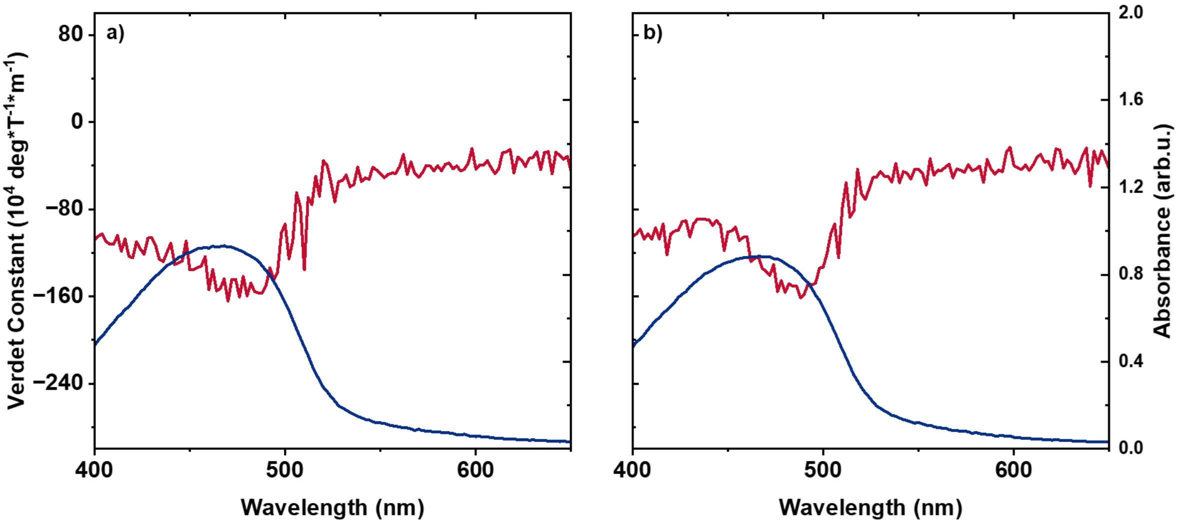

Supplementary Figure 7: Magneto-optical activity (red) of **F8BT:[*M*]-aza[6]H** (a) and **F8BT:[*P*]-aza[6]H** (b) blend films and absorbance spectra (blue) measured using a Mueller-matrix polarimeter and 1.3T permanent magnet. Spectra show the same-sign Verdet constant for both enantiomers, consistent with the measurements using the alternate method in Figure 2d and f of the main manuscript, however the magnitude of the Verdet constants are considerably greater. Film thickness for both film: 220 nm.

Suppl. Note 5. Magnetic field dependence

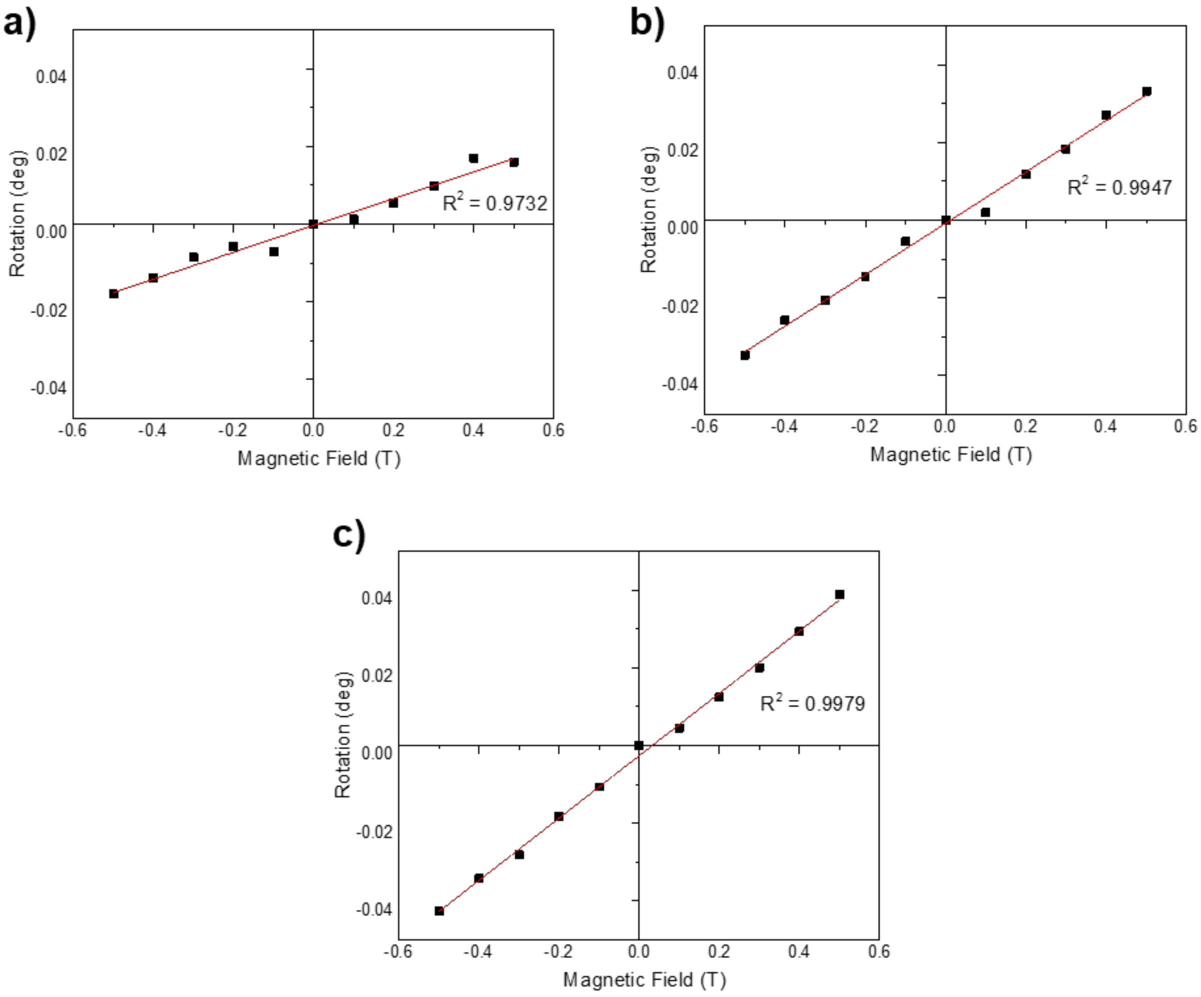

Supplementary Figure 8: Magnetic field vs. rotation dependence of thermally annealed **F8BT:[*P*]-aza[6]H** films at different thicknesses measured at 500 nm. a) 122 nm, b) 216 nm, c) 333 nm.

Suppl. Note 6. Thickness dependency of the Verdet constant and further discussion of the supramolecular structure

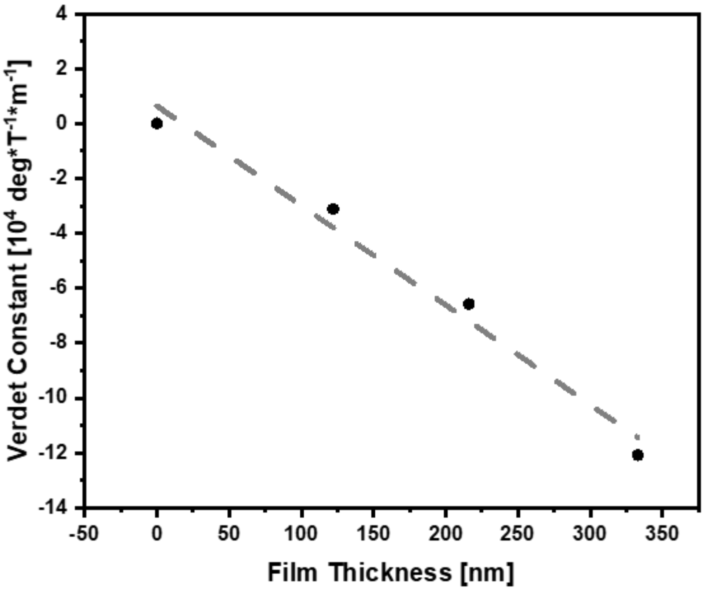

Supplementary Figure 9: Dependence of the Verdet constant vs. the film thickness for **F8BT:10wt% [*P*]-aza[6]H**, thermally annealed, determined at 490 nm.

The supramolecular structure within **F8BT:aza[6]H** films has previously been studied by our group.[4] Specifically, using resonant X-ray scattering we observed an in-plane feature with periodicity on the order of 280nm, whilst high-resolution AFM revealed 25nm wide twisted fibrils. A combination of optical modelling and spectroscopic Mueller matrix ellipsometry ruled out the presence of single domain or multi-domain cholesteric-stack like organisations.[4] The X-ray scattering results are consistent with a weakly ordered “blue-phase”, an arrangement in which cylinders formed from twisted polymer fibrils assemble perpendicular to one another with a periodicity of 280nm (as shown in Figure 1).

In these assemblies, the **aza[6]H** additive acts solely as an inducer of the chiral phase into the otherwise achiral polymer. 10wt% of the helicene is an optimum amount of dopant to achieve this good quality films with a large induced chiroptical response.[5] It is important to note that the measured absorption, natural optical activity and magnetic optical activity originate from the polymer, not the additive.

Supplementary Table 1: Size of supramolecular assemblies in chiral **F8BT:aza[6]H** films.

Feature	***Approximate size***
Polymer fibril	25 nm
Blue-Phase cylinder spacing	260-330 nm
Blue-Phase domain	1-2 µm

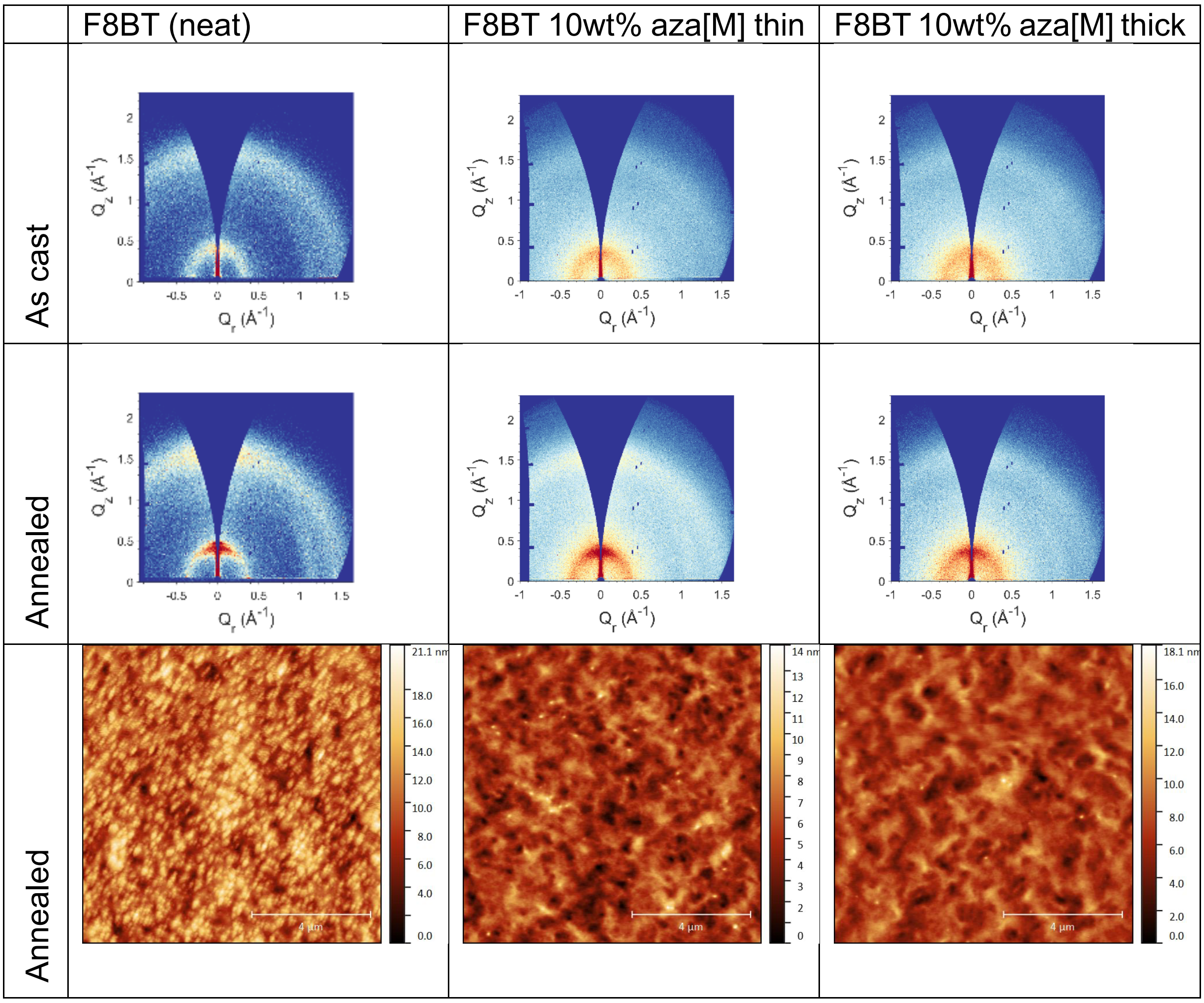

Supplementary Figure 10: GIWAX (first and second row) and AFM (third row) data for as cast (first row) and annealed (second row) neat **F8BT** (first column) as well as thin and thick **F8BT:[*M*]-aza[6]** (second and third column) blends. The colour of the GIWAX images represents the minimum (blue) to maximum (red) recorded intensities.

Suppl. Note 7. Fits to a simple model of excitonically coupled dimers

The Faraday rotation spectra of the **F8BT:[*P*]-aza[6]H** films (122 nm and 333 nm) fit well to simple models of excitonically coupled dimers. These can be compared with Figure 5 in the main manuscript for the 216 nm films.

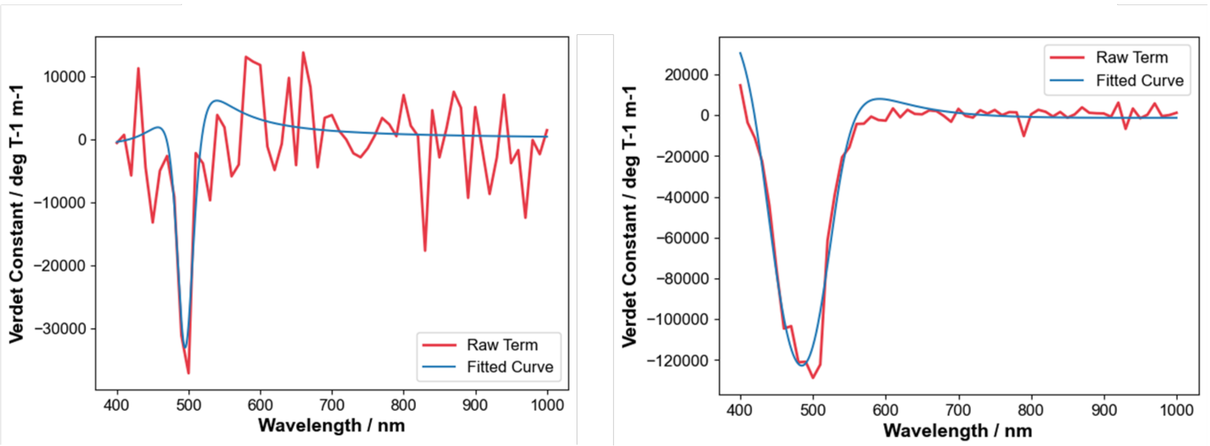

Supplementary Figure 11: Least squares fit to the experimentally observed Faraday spectra for **F8BT:aza[6]** films of the coupled dimers model shown in Figure 4 of the main manuscript. Left: **F8BT:[*P*]-aza[6]H** 122 nm; right: **F8BT:[*P*]-aza[6]H** 333 nm.

Suppl. Note 8. Figure of Merit (FoM) for Faraday rotation

To better evaluate the degree of rotation vs. the absorbance at a given wavelength, we determined the maximum FoM (Supplementary Table 1) achieved for the investigated films. The FoM is defined as Faraday rotation in deg/T divided by the dimensionless absorbance A.[1] Please note that the wavelength of maximum FoM is not necessarily the wavelength of the maximum Verdet constant. For instance, the 333 nm thick **F8BT:[*P*]-aza[6]H** films show a broader magneto-optical response leading to a significant Verdet constant at the slope of the absorbance peak and therefore to a significantly increased maximum FoM.

Supplementary Table 2: Maximum FoM for the investigated film.

Film	Wavelength of maximum FoM [nm]	Absorbance	Verdet constant x 10^4 [°/Tm]	Figure of Merit [°/T]
**F8BT:[*P*]-aza[6]H** *(122 nm)*	500	0.47	-3.7	-0.010
**F8BT:[*P*]-aza[6]H** *(216 nm)*	490	0.77	-6.6	-0.019
**F8BT:[*M*]-aza[6]H** (216 nm)	500	0.74	-7.2	-0.021
**F8BT:[*P*]-aza[6]H** *(333 nm)*	510	0.69	-12.2	-0.059

Suppl. Note 9. Polarization of light after passing through the film

To establish the polarization that is induced into the linearly polarized light after passing through the chiral films we projected a linear Stokes vector through the Mueller matrix data (collected in transmission with the Mueller Matrix Polarimeter (MMP) at Diamond Light Source B23

beamline)[6] recorded for samples at different magnetic fields. This allows us to estimate the polarization vector of the light exiting the sample. ($S_{\mathrm{out}} = \underline{\mathbf{M}}.S_0$).

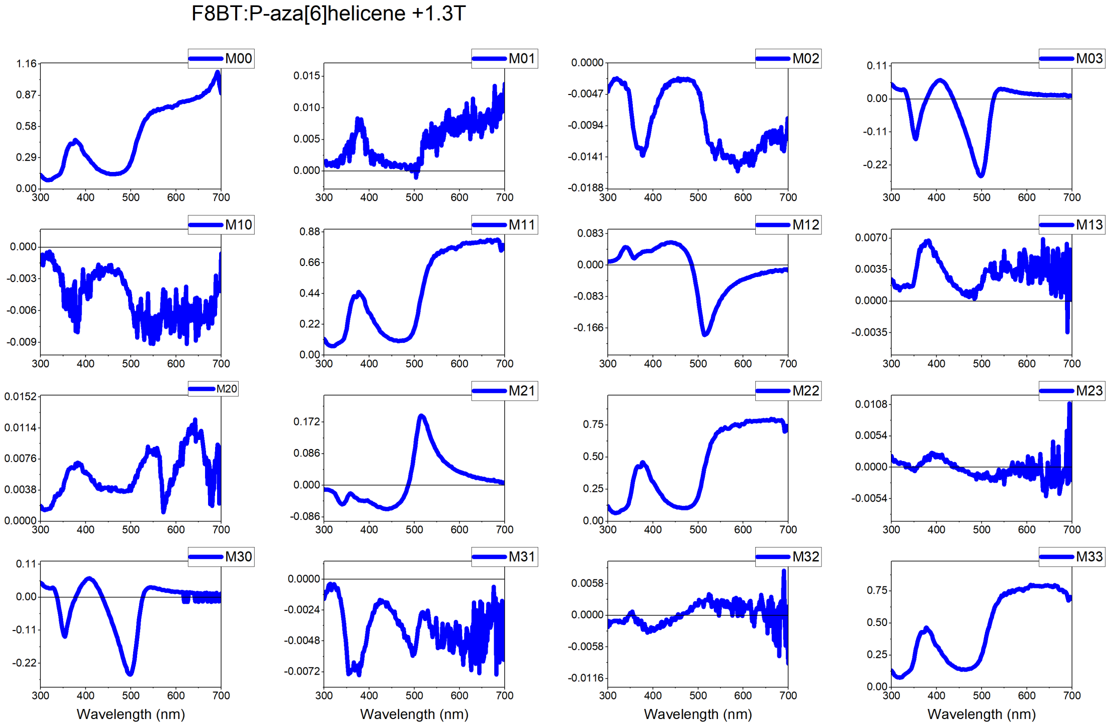

Supplementary Figure 12: Experimental transmission Mueller matrix of an **F8BT:[*P*]-aza[6]H** film recorded in a magnetic field of +1.3T.

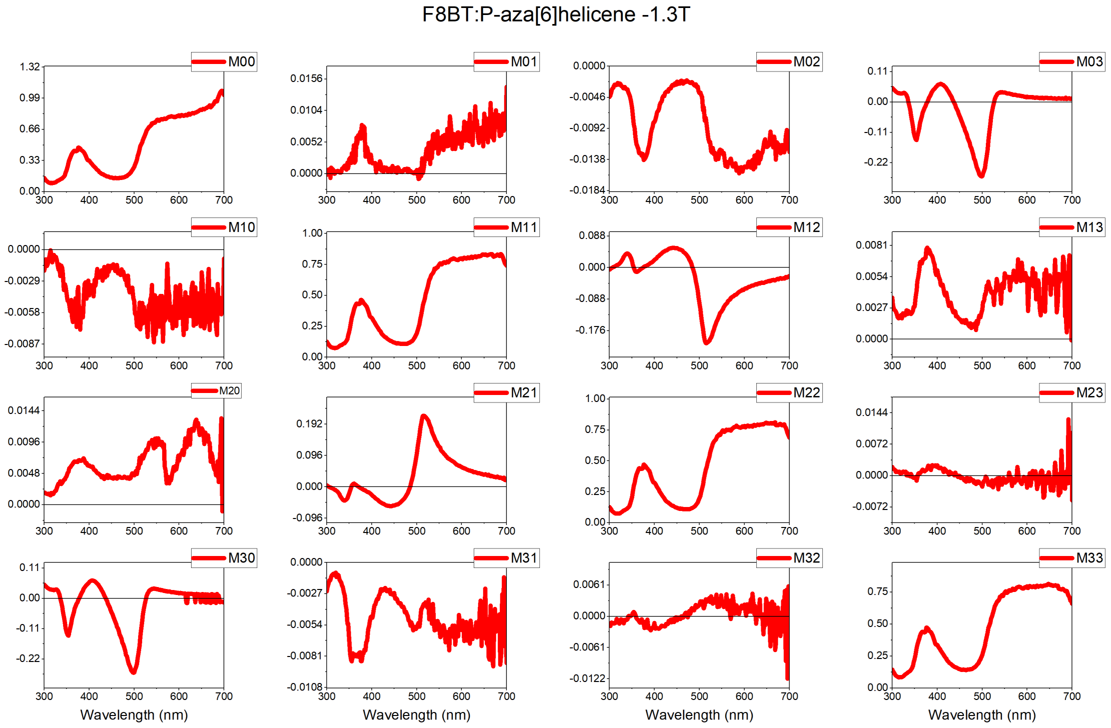

Supplementary Figure 13: Experimental transmission Mueller matrix of an **F8BT:[*P*]-aza[6]H** film recorded in a magnetic field of -1.3T.

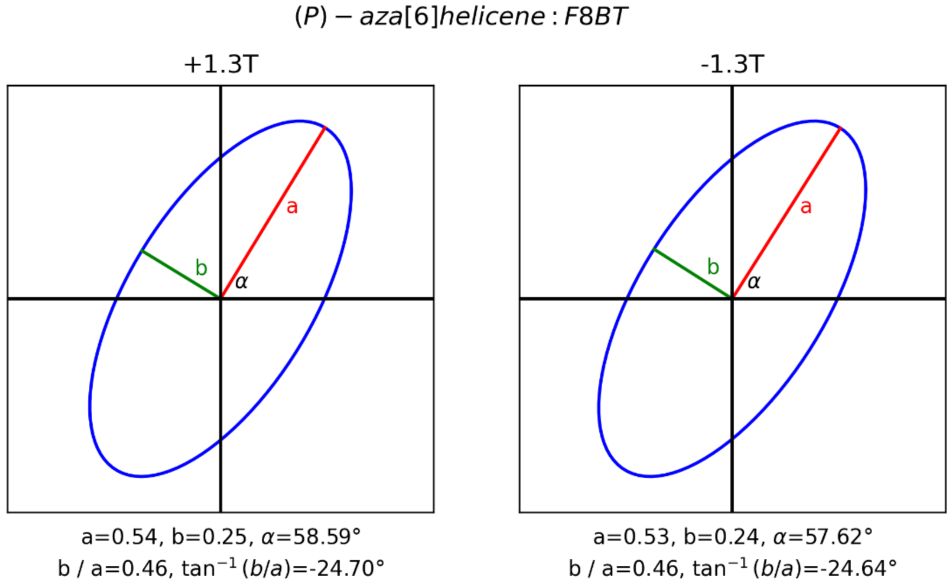

Supplementary Figure 14: Predicted polarization ellipses of 500nm light after transmission through a 220nm thick sample of **F8BT:[*P*]-aza[6]H** in both a positive and negative magnetic field. The ellipses were predicted by multiplying a 45° linearly polarised Stokes vector (S_0=[1,0,1,0]) by the experimentally measured Mueller matrix for the sample at 500nm (S_out=_M.S_0). Ellipses were then visualised using the pypolar package for Python.[7]

Supplementary references

1. Nelson, Z., Delage-Laurin, L., Peeks, M.D., Swager, T.M. Large Faraday Rotation in Optical-Quality Phthalocyanine and Porphyrin Thin Films. *J. Am. Chem. Soc.* **143**,7096-7103 (2021).
2. Delage-Laurin, L., Nelson, Z., Swager, T.M. C-Term Faraday Rotation in Metallocene Containing Thin Films. *ACS Appl. Mater. Interfaces* **13**,25137-25142 (2021).
3. Barron, L.D. Molecular Light Scattering and Optical Activity (Cambridge University Press, UK, 2004). doi: 10.1017/CBO9780511535468
4. Wade, J., Hilfiker, J.N., Brandt, J.R., Liiro-Peluso, L., Wan, L., Shi, X.*, et al.* Natural optical activity as the origin of the large chiroptical properties in pi-conjugated polymer thin films. *Nat. Commun.* **11**,6137 (2020).
5. Wan, L., Wade, J., Wang, X., Campbell, A.J., Fuchter, M.J. Engineering the sign of circularly polarized emission in achiral polymer – chiral small molecule blends as a function of blend ratio. *J. Mater. Chem. C* **10**,5168-5172 (2022).
6. Hussain, R., Javorfi, T., Siligardi, G. CD Imaging at High Spatial Resolution at Diamond B23 Beamline: Evolution and Applications. *Front. Chem.* **9**,616928 (2021).
7. Prahl, S. (2023). pypolar: a python module for polarization using Jones or Mueller calculus (Version 0.9.3) [Computer software]. https://doi.org/10.5281/zenodo8358112
